# Time-of-Day-Specific High-Intensity Interval Training (Chrono-HIIT) in Chinese College Students with Low Physical Activity Levels: Protocol for a Mixed-Methods Feasibility Pilot Randomized Controlled Trial

**DOI:** 10.3390/healthcare14111443

**Published:** 2026-05-23

**Authors:** Wendi Cui, Nor M. F. Farah, Hao Li, Arimi Fitri Mat Ludin

**Affiliations:** 1 Center for Healthy Ageing & Wellness HCARE, Faculty of Health Sciences, Universiti Kebangsaan Malaysia, Kuala Lumpur 50300, Malaysia; 2 Center for Community Health Studies ReaCH, Faculty of Health Sciences, Universiti Kebangsaan Malaysia, Kuala Lumpur 50300, Malaysia

**Keywords:** HIIT, chrono-exercise, circadian rhythm, feasibility pilot trial, physical fitness, college students, randomized controlled trial

## Abstract

Physical inactivity and declining health-related physical fitness among college students are growing global public health concerns. High-intensity interval training (HIIT) is a time-efficient strategy to improve multiple components of health-related physical fitness. Emerging evidence suggests that exercise timing may influence physiological responses and adherence through circadian rhythm regulation; however, its feasibility in college settings, particularly in China, remains unclear. This study aims to evaluate the feasibility and preliminary effectiveness of an eight-week time-specific HIIT programme among Chinese college students, and to compare outcomes between morning and evening training. In this mixed-methods feasibility randomized controlled trial, approximately 72 students with low physical activity levels and intermediate chronotype will be randomly assigned to a morning HIIT group, evening HIIT group, or control group. Intervention groups will complete three HIIT sessions per week for eight weeks. Primary outcomes include feasibility indicators (recruitment, retention, adherence, and data completeness). Secondary outcomes assess changes in body composition, cardiorespiratory fitness, muscular strength, endurance, and flexibility. Quantitative data will be analysed using descriptive and repeated-measures methods, while qualitative interviews will be thematically analysed. Findings will inform the feasibility and design of future large-scale trials and contribute to chrono-exercise research in college populations.

## 1. Introduction

In recent years, declining physical activity levels and deteriorating health-related physical fitness among young adults have become important global public health concerns [1]. College students represent a particularly critical population during the transition from adolescence to adulthood, a period in which long-term lifestyle behaviours, including physical activity patterns, are often established and maintained into later life [2]. However, modern academic environments characterised by increased academic pressure, prolonged sedentary learning, and extensive use of digital technologies have contributed to reduced physical activity participation among college students [3]. Insufficient physical activity in this population has been associated with a range of adverse health outcomes, including reduced cardiovascular fitness, increased risk of obesity, and poorer psychological well-being [4].

In China, the physical health of college students has received growing attention from policymakers and educational institutions [5]. National health surveillance reports have indicated a gradual decline in several health-related physical fitness indicators among Chinese college students, including endurance capacity, muscular strength, and overall participation in physical activity [6]. Although universities have implemented various physical education (PE) programmes and policies to address these issues, the effectiveness of traditional PE curricula has sometimes been limited due to factors such as low student engagement, insufficient exercise intensity, and a lack of innovative teaching approaches [7]. These challenges highlight the need for more efficient and attractive exercise strategies that can be realistically integrated into the daily routines of college students.

High-intensity interval training (HIIT) has emerged as a time-efficient exercise modality that can produce meaningful improvements in cardiorespiratory fitness, metabolic health, and overall physical performance within relatively short training durations [8]. Compared with traditional continuous aerobic exercise, HIIT requires less time commitment while still producing comparable or even superior physiological adaptations, making it particularly suitable for populations facing time constraints, such as university students. Consequently, HIIT-based exercise programmes have increasingly been proposed as practical interventions to promote physical activity participation and improve health-related physical fitness in young adults.

Beyond exercise modality and intensity, increasing attention has recently been directed toward the role of exercise timing in shaping physiological responses and training adaptations. Human physiological processes are regulated by endogenous circadian rhythms—approximately 24-h biological cycles that influence hormone secretion, metabolism, sleep–wake patterns, and physical performance [9]. Emerging evidence suggests that the timing of exercise relative to an individual’s circadian rhythm may influence acute physiological responses, exercise performance, and long-term training outcomes [10]. This concept, often referred to as chrono-exercise, proposes that aligning exercise timing with circadian biological rhythms may enhance training effectiveness and improve adherence to exercise programmes.

These circadian influences may be particularly relevant among college students, whose daily routines, academic schedules, sleep patterns, and physical activity behaviours are often irregular. Previous studies have shown that insufficient physical activity, sleep disruption, and circadian misalignment are associated with poorer physical health outcomes, reduced exercise participation, and lower physical fitness levels among university students [4,11]. Therefore, understanding whether morning or evening exercise produces different responses in this population may help optimize exercise interventions and improve adherence within university settings.

**Figure 1:**
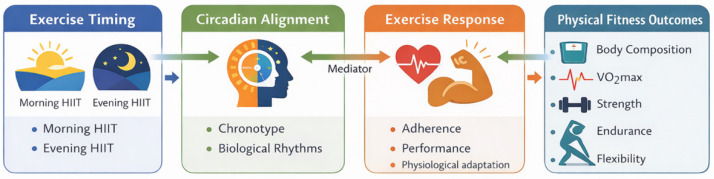
Conceptual framework of the time-specific high-intensity interval training (HIIT) intervention.

Individual differences in circadian rhythm are commonly described through chronotype, which reflects a person’s natural preference for earlier or later timing of daily activities and sleep patterns. Individuals may be classified as morning types, evening types, or intermediate types based on their circadian preference. Previous studies have suggested that chronotype may influence physical performance, motivation to exercise, and adherence to structured training programmes [11]. Consequently, the timing of exercise sessions may interact with circadian biology to influence both physiological outcomes and behavioural responses to exercise interventions. Figure 1, developed based on existing chrono-exercise and circadian rhythm literature [10,12,13], illustrates the proposed mechanistic pathway through which exercise timing (morning vs. evening HIIT) may interact with circadian alignment factors, including chronotype and endogenous biological rhythms, to influence exercise-related responses such as adherence, physical performance, and physiological adaptation. These responses are expected to contribute to improvements in health-related physical fitness outcomes, including body composition, cardiorespiratory fitness, muscular strength, muscular endurance, and flexibility.

Despite the growing interest in chrono-exercise, empirical evidence examining the feasibility and effectiveness of time-specific exercise interventions in university settings remains limited. Few studies have explored whether performing HIIT at different times of day such as morning versus evening may produce different health-related physical fitness outcomes or influence participants’ perceptions and experiences of the training programme. Understanding these factors is important for designing exercise interventions that are both physiologically effective and practically acceptable within real-world educational environments.

To address this research gap, the present study developed a structured HIIT module specifically designed for Chinese college students. Before implementing large-scale intervention programmes, it is essential to evaluate the feasibility and practicality of the training module within the target population. Feasibility studies play a critical role in the early stages of intervention development by assessing recruitment, adherence, acceptability, and implementation challenges within real-world contexts [14]. In addition to quantitative assessments of health-related physical fitness outcomes, qualitative methods such as semi-structured interviews can provide valuable insights into participants’ experiences, perceived benefits, and potential barriers related to the intervention.

Therefore, the present study aims to evaluate the feasibility and preliminary effect of an eight-week time-specific high-intensity interval training (Chrono-HIIT) programme among low-physically active Chinese college students with an intermediate chronotype. The primary objective of this feasibility pilot randomized controlled trial is to assess the practicality and acceptability of implementing the intervention, including participant recruitment, retention, adherence, safety, and data completeness. The secondary objective is to explore the preliminary effects of morning versus evening HIIT on health-related physical fitness outcomes, including body composition, cardiorespiratory fitness, muscular strength, muscular endurance, and flexibility. In addition, the qualitative component of the study aims to explore participants’ experiences, perceived benefits, barriers to participation, and overall acceptability of the intervention. It is hypothesized that the Chrono-HIIT programme will demonstrate acceptable feasibility and adherence among low-physically active Chinese college students and may produce preliminary improvements in health-related physical fitness outcomes. The findings may contribute to the optimization of physical education programmes and inform the design of future large-scale randomized controlled trials aimed at promoting efficient and sustainable physical activity interventions in college settings.

## 2. Materials and Methods

### 2.1. Study Design

This study is designed as a feasibility pilot randomized controlled trial, consistent with CONSORT extension guidelines for pilot and feasibility studies consisting of two sequential phases: (1) a quantitative feasibility trial and (2) a qualitative exploration of participant experiences with the intervention. The protocol has been developed and reported in accordance with the Standard Protocol Items: Recommendations for Interventional Trials (SPIRIT) guidelines, which provide a standardized framework for reporting clinical trial protocols.

The study will be conducted at Qiqihar University, Qiqihar, China. In Phase I, eligible participants will be randomly allocated to one of three groups: Morning HIIT, Evening HIIT, or Control group, using a restricted lottery-based randomisation method with allocation concealment. Randomisation will be conducted after eligibility confirmation, written informed consent, and completion of baseline assessments. Identical opaque sealed envelopes containing group assignments will be prepared by an independent researcher and selected by participants at the point of allocation. Participants in the intervention groups will complete an eight-week high-intensity interval training (HIIT) programme, with the primary distinction between groups being the timing of the training sessions (morning or evening). Participants in the control group will continue their usual daily routines without participating in the structured exercise programme during the study period.

Due to the nature of the exercise intervention, participants and exercise instructors cannot be blinded to group allocation. Therefore, the present study will adopt an assessor-blinded design. Outcome assessors and data analysts will remain blinded to participant allocation where feasible to minimize potential assessment and analytical bias throughout the study period.

Phase II will involve a qualitative investigation aimed at exploring participants’ experiences with the intervention. A purposive sample of participants who completed the intervention will be invited to participate in semi-structured interviews to explore perceptions of the HIIT programme, including its feasibility, acceptability, perceived benefits, and potential barriers to participation.

Approval to conduct the study at Qiqihar University was obtained prior to participant recruitment and data collection. This approval was granted through the Qiqihar University Experimental Application Form, dated 6 June 2024, with institutional endorsement provided on 10 June 2024. The document did not include a formal approval reference number. As the study forms part of a doctoral research project undertaken under the academic supervision of Universiti Kebangsaan Malaysia, formal research ethics approval was subsequently obtained from the Universiti Kebangsaan Malaysia Research Ethics Committee (Approval No. JEP-2025-206). The study was also registered with the Thai Clinical Trials Registry (Registration No. TCTR20250607005). All participants will provide written informed consent before enrolment, and the study will be conducted in accordance with the principles of the Declaration of Helsinki.

### 2.2. Pilot Trial Justification

Feasibility studies are recommended prior to conducting definitive randomized controlled trials to assess whether an intervention can be successfully implemented in real-world settings [14]. Such studies help identify potential issues related to participant recruitment, adherence, intervention delivery, and outcome measurement, which may influence the success of larger-scale trials.

In the context of exercise interventions among college students, feasibility studies are particularly important due to potential barriers such as academic schedules, time constraints, and variability in exercise adherence. The present study therefore aims to evaluate the practicality, acceptability, and preliminary effectiveness of a time-specific HIIT programme for Chinese college students. Findings from this pilot trial will inform the refinement of the intervention protocol and support the design of a future fully powered randomized controlled trial.

### 2.3. Sample Size

Sample size estimation for the pilot trial was guided by the recommendations of Whitehead et al., [15] for pilot randomized trials. These recommendations indicate that pilot studies should recruit sufficient participants to provide preliminary estimates of effect size that can inform the sample size calculation for a subsequent definitive trial.

Based on these guidelines, approximately 15–25 participants per group are considered adequate for pilot trials investigating continuous outcomes with moderate effect sizes. Therefore, the present study aims to recruit approximately 72 participants in total, with 24 participants allocated to each study group (morning HIIT, evening HIIT, and control).

For the qualitative component, interview-based studies typically achieve data saturation with 9–17 participants [16]. Therefore, approximately 10–15 participants will be purposively selected for interviews. Additional interviews will be conducted if new themes continue to emerge during analysis.

### 2.4. Eligibility Criteria

Participants will be recruited from full-time undergraduate students enrolled at Qiqihar University. To be eligible for inclusion, participants must be full-time undergraduate students at Qiqihar University, classified as having an intermediate chronotype as determined by the Chinese version of the Morningness–Eveningness Questionnaire (MEQ) [17], and identified as having low physical activity levels (<600 MET-min/week) based on the short form of the International Physical Activity Questionnaire (IPAQ) [18]. Both the Chinese version of the MEQ (Cronbach’s α= 0.769) and the IPAQ-SF (Cronbach’s α = 0.82) are widely used instruments that have demonstrated acceptable reliability and validity in Chinese populations and have been extensively applied in health- and physical activity-related research.

Participants will be excluded if they are unable to safely participate in physical activity, have clinically diagnosed mental disorders, musculoskeletal or cardiovascular conditions that contraindicate high-intensity exercise participation, or are unable to provide informed consent. Prior to participation, all eligible participants will undergo basic health screening to ensure the safety and appropriateness of participation in the HIIT intervention programme.

### 2.5. Recruitment Procedures

Participants will be recruited through announcements distributed across the university campus. Interested students will first complete screening questionnaires including the MEQ and IPAQ. MEQ scores between 42 and 58 will classify participants as having an intermediate chronotype, while IPAQ scores below 600 MET-min/week will indicate low physical activity levels. Students meeting both criteria will be invited to participate in the study.

### 2.6. Randomization and Allocation

Eligible participants will be randomly assigned to one of three groups: Morning HIIT, Evening HIIT, or Control group, using a restricted lottery-based randomisation method with a 1:1:1 allocation ratio. To generate the allocation sequence, a total of 72 identical opaque sealed envelopes will be prepared, comprising 24 envelopes labelled “Morning HIIT”, 24 envelopes labelled “Evening HIIT”, and 24 envelopes labelled “Control”. The envelopes will be identical in size, colour, and appearance, and each allocation label will be folded and sealed inside the envelope so that the assignment cannot be seen before selection.

To ensure allocation concealment, the envelopes will be prepared and mixed by an independent researcher who is not involved in participant recruitment, baseline assessment, intervention delivery, or outcome assessment. The sealed envelopes will be placed in an opaque container and thoroughly mixed before the allocation process begins. Randomisation will be conducted only after eligibility confirmation, written informed consent, and completion of baseline assessments.

At the point of allocation, each participant will independently draw one sealed envelope from the opaque container under the supervision of a research team member. The selected envelope will then be opened to reveal the participant’s group assignment. Once selected, the envelope will not be returned to the container. This lottery procedure will be conducted without replacement until the required allocation of 24 participants per group is achieved. This process ensures that each participant’s allocation is concealed before assignment and is made independently and unpredictably upon enrolment.

**Figure 2:**
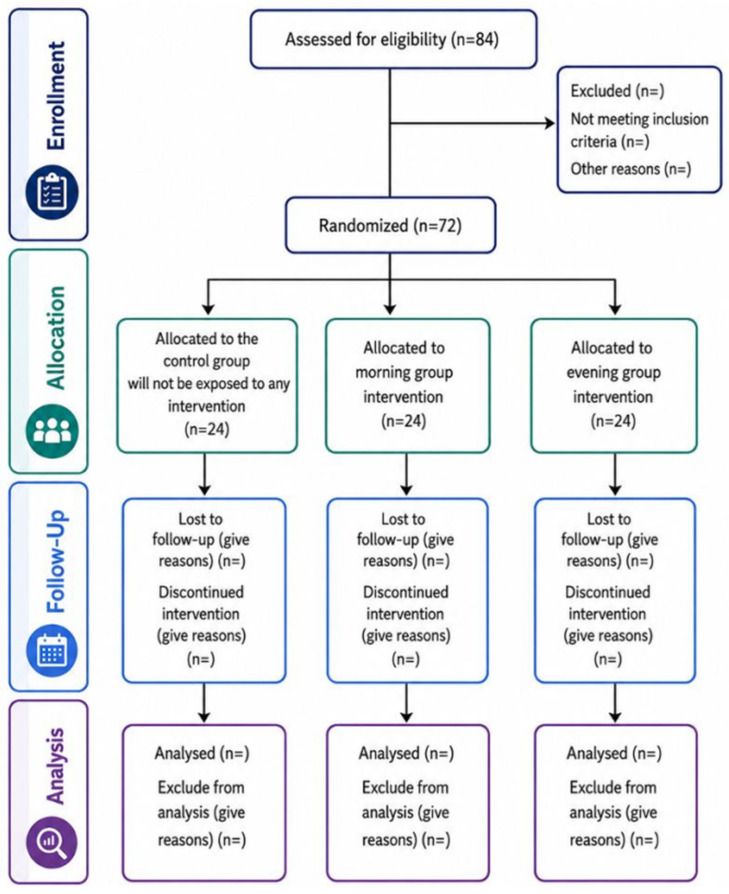
CONSORT Flow Diagram for the Chrono-HIIT Study.

Participants allocated to the Morning HIIT group will perform the training sessions in the morning, whereas participants allocated to the Evening HIIT group will perform identical training sessions in the evening. Participants allocated to the Control group will maintain their usual daily routines without participating in structured exercise during the study period. A schematic CONSORT diagram illustrating the intervention procedure is shown in Figure 2. Table 1 outlines the participant recruitment timeline, key milestones, and planned assessment activities.

### 2.7. Intervention

#### Chrono-HIIT Exercise Intervention Protocol

The Chrono-HIIT intervention programme was developed specifically for college student populations, taking into consideration their general fitness levels, feasibility within a college setting, and the need for a time-efficient exercise strategy. The programme is designed as a short-duration body weight high-intensity interval training (HIIT) session that can be performed without specialised equipment. Each training session comprises three sequential phases: warm-up, HIIT, and cool-down, with a total session duration of approximately 10 min.

*Warm-up Phase*

A structured warm-up is performed at the beginning of each session to prepare the musculoskeletal and cardiovascular systems for subsequent high-intensity activity and to reduce the risk of exercise-related injury [19]. Participants will begin with 60 s of light jogging in place, which gradually elevates heart rate and increases blood circulation to the working muscles.

This will be followed by dynamic stretching exercises involving both upper and lower extremities, including movements targeting the shoulders, trunk, hips, and lower limbs. These dynamic movements are intended to improve joint mobility, enhance neuromuscular activation, and ensure physiological readiness before commencing the high-intensity exercise phase.

*HIIT Phase*

The HIIT component consists of eight simple body-weight movements designed to be easily performed by college students without specialised equipment. The total duration of the HIIT phase is approximately 8 min, with exercises performed in repeated high-intensity bouts interspersed with brief recovery periods. The detailed exercise protocol, including the sequence of movements and interval duration, is presented in Table 2.

Exercise intensity during the HIIT phase will target approximately 80–90% of the participants’ age-predicted maximum heart rate (HRmax), which is consistent with recommended intensity ranges for high-intensity interval training in young adults. Estimated HRmax will be calculated using the age-predicted formula: HRmax = 220-age [20]. Prior to the intervention, the principal investigator will provide standardised demonstrations of all exercise movements to ensure correct technique and consistency across participants. Training sessions will only begin once participants confirm their understanding of the exercises and demonstrate the ability to perform them safely and correctly.

To ensure intervention fidelity and adherence, attendance will be recorded for each training session. Participants will also be verbally encouraged to maintain the required exercise intensity during the intervals. Any adverse events, discomfort, or injuries occurring during the training sessions will be documented and monitored throughout the intervention period to ensure participant safety.

*Cool-down Phase*

Following completion of the HIIT session, participants will perform a brief cool-down phase consisting of relaxation exercises and static stretching. The primary aim of the cool-down is to gradually reduce cardiovascular demand and facilitate physiological recovery after high-intensity activity [21].

The cool-down routine includes static stretching exercises targeting the major muscle groups involved during the training session, particularly the lower extremities and upper body. These stretches help relieve muscle tension, restore normal muscle length, and improve flexibility following vigorous exercise [22].

Overall, the cool-down phase is designed to promote post-exercise recovery and minimise the likelihood of delayed onset muscle soreness or exercise-related discomfort.

### 2.8. Exercise Intensity Monitoring

Exercise intensity will be monitored using both objective and subjective measures. For an objective measure, participants will wear heart rate monitoring wristbands during all training sessions. Exercise intensity will be maintained at approximately 80–90% of estimated maximal heart rate (HRmax). While for subjective measure, participants will also rate their perceived exertion using the modified Borg Rating of Perceived Exertion (RPE) scale (0–10) immediately after each training session.

Given that the study involves participants with low prior physical activity levels, safety monitoring will be conducted throughout the intervention period. Before each training session, participants will be asked about their current physical condition, including any musculoskeletal pain, illness, dizziness, unusual fatigue, or other symptoms that may affect safe participation. During each session, the principal investigator or trained research personnel will observe participants for signs of exercise intolerance, including excessive breathlessness, dizziness, chest discomfort, nausea, musculoskeletal pain, or inability to maintain correct movement technique. Participants will be instructed to stop exercising immediately if they experience any concerning symptoms.

All adverse events and safety-related incidents will be documented using a standardized adverse event recording form. Information recorded will include the type of event, timing, severity, relationship to the intervention, action taken, and outcome. Serious adverse events, if any, will be reported to the research team and relevant ethics committee in accordance with institutional requirements.

### 2.9. Outcome Measure

#### 2.9.1. Feasibility Outcomes

The primary outcomes of the study are feasibility indicators, including recruitment rate, retention rate, adherence rate and data missing rate. Recruitment will be considered successful if more than 70% of eligible students agree to participate. Retention will be considered acceptable if more than 80% of participants complete the intervention. Adherence will be defined as participation in more than 70% of the prescribed training sessions. To account for partial participation, adherence will also be calculated as the proportion of completed training sessions relative to the total number of prescribed sessions. Participants who attend some, but not all, scheduled sessions will therefore be categorized according to their overall attendance percentage. The overall missing data rate is expected to remain below 10% [23,24]. Feasibility thresholds were established based on recommendations for pilot and feasibility trials and commonly used progression criteria in feasibility research [25].

Safety-related outcomes will include the number, type, severity, and relatedness of adverse events occurring during the intervention period. Adverse events will include any undesirable physical or psychological symptoms occurring during or after the HIIT sessions, such as musculoskeletal pain, dizziness, excessive fatigue, nausea, shortness of breath beyond expected exertion, or injury. Events will be categorized as mild, moderate, or severe, and as unrelated, possibly related, or probably related to the intervention. These safety indicators will be used to evaluate whether the Chrono-HIIT programme can be delivered safely among college students with low prior physical activity levels.

#### 2.9.2. Health Related Physical Fitness Outcomes

All health-related physical fitness and body composition measurements will be conducted by trained research personnel following standardized testing procedures to ensure consistency and reliability of the measurements. Prior to data collection, assessors will receive training on the testing protocols and equipment use.

All assessments will be conducted indoors under controlled environmental conditions to minimize external influences on performance. To reduce potential confounding factors, participants will be instructed to avoid vigorous physical activity, caffeine intake, and heavy meals for at least two hours prior to testing.

To minimize fatigue effects and ensure consistency, the tests will be conducted in the following standardized order: body composition assessment, cardiorespiratory fitness test, muscular strength tests, muscular endurance tests, flexibility assessment. Adequate rest intervals of 3–5 min between tests will be provided to allow sufficient recovery and prevent fatigue from influencing subsequent test performance.

The study will generate preliminary estimates of intervention effects on several health-related physical fitness outcomes. Although the study is not powered to detect definitive intervention effects, the results will provide initial estimates of effect size that can inform sample size calculations for future large-scale randomized controlled trials.

*Body Composition Measurement*

Body mass index (BMI) will be calculated using the standard formula:

$$\mathrm{BMI}\;=\;\frac{\mathrm{weight}\;(\mathrm{kg})}{{\mathrm{height}\;(m)}^{2}}$$

Participants’ height and body weight will be directly measured by trained researchers using standardized equipment, which will be used to compute BMI values. Height will be measured using an electronic stadiometer (iboga C8), while body weight will be measured using a body composition analyzer (InBody Dial H20N). BMI is widely applied in epidemiological studies as an indicator of weight status and has been shown to be strongly associated with total body fat [26]. Based on the calculated BMI values, participants will be categorized into four weight status groups: underweight (<18.5 kg/m^2^), normal weight (18.5–23.9 kg/m^2^), overweight (24–27.9 kg/m^2^), and obese (≥28 kg/m^2^).

In addition to BMI, body fat percentage and skeletal muscle mass will be assessed using a bioelectrical impedance analyzer (InBody H20, InBody Co., Ltd., Seoul, Republic of Korea). Prior to the assessment, participants will be instructed to remove all metal accessories, such as earrings, bracelets, and watches, to avoid potential interference with the electrical current used by the device.

Measurements will be performed when participants are in a normal physiological state, avoiding assessment immediately after food intake or vigorous physical activity. During the measurement procedure, participants will stand barefoot on the analyzer platform with both feet fully contacting the electrode plates. They will hold the device handles with arms relaxed at their sides to ensure proper contact between the fingers and electrodes, allowing accurate measurement of body composition parameters.

*Cardiorespiratory Fitness Assessment*

Cardiorespiratory fitness will be assessed using the Queen’s College Step Test, a validated and widely used submaximal field test for estimating maximal oxygen uptake (VO_2_max) in young adults [27]. The test is suitable for field-based studies due to its simplicity, safety, and minimal equipment requirements.

Participants will perform the step test on a 41.3 cm (16.25 inch) step bench for 3 min. The stepping cadence will be controlled using a metronome, set at 24 steps per minute for males and 22 steps per minute for females, following the standardized protocol. Participants will be instructed to maintain a consistent stepping rhythm using the sequence up–up–down–down throughout the test.

Immediately after completing the step test, participants will sit down, and their recovery heart rate will be measured between 5 and 20 s during the recovery period using a heart rate monitor. The recorded heart rate will then be converted to beats per minute (bpm) for analysis.

Estimated VO_2_max (ml·kg^−1^·min^−1^) will be calculated using the following standard prediction equations:

For males:

VO_2_max = 111.33 − (0.42 × heart rate)

For females:

VO_2_max = 65.81 − (0.1847 × heart rate)

Higher estimated VO_2_max values indicate greater cardiorespiratory fitness capacity.

*Muscular Strength Assessment*

Upper- and lower-body muscular strength will be assessed using one-repetition maximum (1RM) tests for the chest press and leg press, respectively. The 1RM test is a widely accepted method for evaluating maximal dynamic muscular strength and has demonstrated good reliability in healthy young adults [28]. Both tests will be conducted using fixed resistance machines, thereby minimizing movement instability and reducing the risk of exercise-related injury. Prior to formal testing, participants will attend a familiarization trial to practice the testing procedures and movement techniques. Participants will be screened for musculoskeletal contraindications before maximal strength testing. All tests will be supervised by trained personnel to ensure proper lifting technique and participant safety.

Before testing, participants will complete a standardized warm-up consisting of light aerobic activity followed by dynamic stretching to prepare the muscles for maximal effort.

For the chest press test, participants will perform the exercise using a resistance machine while maintaining proper body alignment and controlled movement technique. For the barbell back squat, participants will stand with feet shoulder-width apart and descend until the thighs are approximately parallel to the floor, while maintaining correct posture and spinal alignment.

The testing load will be progressively increased until the participant can complete only one repetition with proper technique, which will be recorded as the 1RM value. Participants will be given 3–5 min of rest between attempts to minimize fatigue. In most cases, the maximal strength value will be determined within three to five attempts.

If a participant fails to complete a repetition with proper technique, the load will be reduced following an adequate rest period and another attempt will be performed. The highest successfully lifted load (kg) will be recorded as the participant’s upper-body strength (chest press 1RM) and lower-body strength (squat 1RM).

*Muscular Endurance Assessment*

The Arm Hang Test measures upper-body muscular endurance by recording the duration (in seconds) that participants can maintain a chin-above-bar position while hanging from a horizontal bar with elbows flexed at approximately 90 degrees. Participants will be instructed to keep their body as still as possible during the test. Timing will begin once the participant assumes the correct position and will stop when the chin drops below the bar or when the participant can no longer maintain proper form.

Lower-body muscular endurance will be evaluated using the Wall Sit Test. Participants will lean against a wall and descend into a seated position with knees flexed at approximately 90 degrees and thighs parallel to the floor. Participants will be asked to maintain the position for as long as possible while keeping their back against the wall. The duration (in seconds) that participants can sustain the position without breaking form will be recorded. Longer durations in each test indicate greater muscular endurance capacity.

*Flexibility Assessment*

Flexibility will be assessed using the Sit-and-Reach Test, which evaluates the flexibility of the hamstring muscles and lower back [29].

Participants will sit on the floor with legs fully extended and knees kept straight, with the soles of their feet placed flat against the sit-and-reach box. With hands placed one on top of the other and arms extended forward, participants will slowly reach forward as far as possible along the measuring scale.

The distance reached (cm) will be recorded as the flexibility score, with greater reach distances indicating better flexibility. Each participant will perform the test twice, and the highest recorded value will be used for analysis.

### 2.10. Qualitative Interviews

Semi-structured interviews will be conducted with a subset of participants from the intervention groups to explore their perceptions and experiences regarding the HIIT module [30]. Semi-structured interviews allow participants to describe their experiences in depth while enabling the researchers to systematically explore key aspects of the intervention.

The interview questions will be developed based on previous qualitative research examining exercise intervention experiences [31]. The interview guide will focus on several core domains, including participants’ overall experiences with the intervention, perceived physical responses, psychological effects, perceptions of the training design, and suggestions for improving the programme. The detailed interview guide is presented in Table 3.

Participant recruitment for the interviews will follow the data saturation approach described by Francis [32]. Initially, 10 participants will be interviewed. Following the completion of these interviews, the data will be analysed to determine whether new themes continue to emerge. If additional themes are identified, three further interviews will be conducted. This process will continue in increments of three participants until no new themes emerge from the data, indicating that thematic saturation has been achieved.

To ensure a balanced representation of perspectives, the study will also aim to maintain gender balance among interview participants, targeting an approximate 1:1 male-to-female ratio. This approach is intended to account for potential gender-related differences in exercise perceptions and experiences [33].

Prior to the interviews, participants will be provided with clear information regarding the purpose of the study, interview procedures, and the intended use of the collected data. Written or verbal informed consent will be obtained before participation. Participants will also be informed that their participation is voluntary and that they may withdraw from the interview at any time without any negative consequences.

To ensure confidentiality and protect participant privacy, strict data protection procedures will be implemented. All interview recordings, transcripts, and related materials will be securely stored, and participants’ identities will be anonymized using unique identification codes rather than personal names.

### 2.11. Data Management

All participants will be assigned unique identification codes to ensure confidentiality. Personal identifying information will not be included in the analytical dataset. All data will be securely stored and accessible only to the research team.

### 2.12. Statistical Analysis

All statistical analyses will be conducted using IBM SPSS Statistics version 27.0 (IBM Corp., Armonk, NY, USA). Descriptive statistics will be used to summarize participant characteristics, feasibility outcomes, and safety indicators. Continuous variables will be presented as means and standard deviations for normally distributed data, while medians and interquartile ranges will be reported for non-normally distributed data. Categorical variables will be presented as frequencies and percentages.

Prior to inferential analyses, the distribution of continuous variables will be assessed using the Shapiro–Wilk test and graphical inspection methods, including histograms and Q–Q plots. Homogeneity of variance and sphericity assumptions will also be evaluated where appropriate. Mauchly’s test of sphericity will be performed for repeated-measures analyses, and Greenhouse–Geisser corrections will be applied if the assumption of sphericity is violated.

As this study is designed as a feasibility pilot randomized controlled trial, analyses of intervention effects will be considered exploratory in nature. Therefore, greater emphasis will be placed on estimating effect sizes and confidence intervals rather than relying solely on statistical significance testing. Mixed designed ANOVA will be used to explore the effects of time (pre-intervention vs. post-intervention), group (morning HIIT, evening HIIT, and control), and time × group interactions on health-related physical fitness outcomes.

If substantial deviations from normality are observed, appropriate remedial approaches such as data transformation or robust statistical procedures will be considered. In addition, non-parametric analyses may be conducted as sensitivity analyses to evaluate the robustness of the findings.

Effect sizes will be calculated to provide preliminary estimates of intervention effects and to inform sample size estimation for future fully powered randomized controlled trials. Statistical significance will be set at *p* < 0.05. Where appropriate, intention-to-treat analyses will be performed to account for missing data and participant attrition.

## 3. Discussion

This study presents the protocol for a mixed-methods feasibility randomized controlled trial designed to evaluate the feasibility and preliminary effectiveness of a time-specific high-intensity interval training (HIIT) programme for improving health-related physical fitness among Chinese college students. By combining quantitative assessments of physical fitness with qualitative exploration of participant experiences, the study aims to provide a comprehensive evaluation of both the practical implementation and perceived acceptability of the intervention in a university setting.

Physical inactivity among college students has become an increasing public health concern globally. Several studies have reported that college students often experience reduced physical activity levels due to academic workload, sedentary learning environments, and competing time demands [34]. Insufficient physical activity during this life stage may have long-term implications for cardiovascular health, metabolic function, and overall well-being [35]. Consequently, developing efficient and accessible exercise interventions that can be realistically integrated into students’ daily schedules is an important priority for public health and university health promotion initiatives.

High-intensity interval training (HIIT) has gained considerable attention as a time-efficient exercise strategy capable of producing meaningful improvements in cardiorespiratory fitness and metabolic health within relatively short training durations [8]. Compared with traditional continuous aerobic training, HIIT requires substantially less time commitment while still eliciting significant physiological adaptations. This makes HIIT particularly attractive for university students who frequently report lack of time as a major barrier to exercise participation. Previous studies have demonstrated that brief HIIT interventions can significantly improve cardiorespiratory fitness, body composition, and metabolic markers in young adults [36,37], further supporting the potential value of HIIT-based programmes for this population.

Another important feature of the present study is the investigation of exercise timing, an emerging topic within exercise physiology and chronobiology. Human physiological processes are regulated by endogenous circadian rhythms that influence hormonal secretion, metabolism, sleep–wake patterns, and physical performance [9]. Growing evidence suggests that the timing of exercise relative to these circadian rhythms may influence both acute physiological responses and long-term training adaptations [10]. This concept, often referred to as chrono-exercise, proposes that aligning exercise timing with circadian biological rhythms may optimize exercise outcomes and potentially enhance adherence to physical activity programmes [13].

Individual differences in circadian rhythm are commonly described through the concept of chronotype, which reflects a person’s natural preference for earlier or later timing of daily activities. Chronotype has been shown to influence exercise performance, motivation, and behavioural responses to physical activity [11]. For example, individuals with morning chronotypes often demonstrate higher physical performance earlier in the day, whereas evening chronotypes may perform better later in the day. Understanding how exercise timing interacts with circadian biology may therefore help optimize training effectiveness and improve adherence to structured exercise programmes [12].

Despite increasing interest in chrono-exercise, empirical research examining the feasibility of time-specific exercise interventions in real-world university settings remains limited. Many previous studies investigating exercise timing have been conducted in controlled laboratory environments or among trained athletes, which may limit their applicability to broader populations such as university students. Therefore, the present study addresses an important gap in the literature by examining whether implementing a structured HIIT programme at different times of day is feasible and acceptable within a university context.

Another strength of the present study is the mixed-methods design, which integrates quantitative and qualitative approaches to evaluate the intervention. While quantitative assessments will provide objective evidence regarding changes in health-related physical fitness indicators, the qualitative interviews will allow participants to describe their experiences with the intervention in greater depth. Understanding participants’ perspectives, including perceived benefits, barriers, and motivational factors, is essential for refining exercise interventions and improving their long-term sustainability. Mixed-methods approaches have increasingly been recommended in feasibility studies because they provide richer insights into intervention implementation and contextual factors influencing participant engagement [14].

The findings of this feasibility trial are expected to contribute to several important areas of research. First, the study will provide evidence regarding the practical implementation of short-duration HIIT programmes within university settings, including recruitment feasibility, participant adherence, and intervention acceptability. Second, the study will generate preliminary data on whether the timing of exercise sessions may influence training responses among university students. Such findings may help inform the design of future chrono-exercise interventions and support the development of more individualized physical activity recommendations.

Nevertheless, several limitations should be acknowledged. The present study only includes participants with an intermediate chronotype in order to reduce circadian variability and improve internal consistency during this preliminary feasibility trial. However, this approach may limit the generalizability of the findings to individuals with extreme morning or evening chronotypes. Therefore, the study does not allow examination of potential chronotype and exercise timing interactions. And the sample size is relatively small; the study may not be powered to detect definitive intervention effects. The study will also be conducted at a single university, which may limit the generalizability of the findings to other student populations or cultural contexts. Because the intervention targets students with low prior physical activity levels, systematic monitoring of adverse events and safety indicators will provide important information on whether short-duration HIIT can be implemented safely and acceptably in this population before proceeding to a larger definitive trial.

Additionally, the intervention duration of eight weeks may not capture long-term behavioural changes or sustained improvements in health-related physical fitness. Future studies may therefore consider multi-site trials, larger sample sizes, and longer intervention periods to further evaluate the effectiveness of time-specific exercise interventions. And future large scale randomized controlled trials should consider including multiple chronotype categories and examining chronotype as a moderating variable influencing exercise adherence and physiological adaptation to time-specific HIIT interventions.

Future research could also expand the scope of investigation by examining additional outcomes such as sleep quality, psychological well-being, and metabolic biomarkers, which are closely linked to circadian physiology and physical activity behaviour. Furthermore, integrating wearable technology and digital health monitoring may provide valuable insights into real-world exercise behaviour and adherence patterns among university students [38].

In summary, this study aims to provide preliminary evidence regarding the feasibility and potential effectiveness of a time-specific HIIT intervention among Chinese college students. The findings are expected to inform the development of future large-scale randomized controlled trials and contribute to the growing field of chrono-exercise research, ultimately supporting the design of more effective and sustainable physical activity interventions for young adult populations.

## 4. Conclusions

This protocol describes a mixed-methods feasibility randomized controlled trial designed to examine the feasibility and potential effectiveness of a HIIT module for improving health-related physical fitness among Chinese college students. The study will also explore the influence of exercise timing and participants’ experiences with the intervention. The findings will provide preliminary evidence to inform the design of future large-scale trials and support the development of practical and time-efficient physical activity interventions in university settings.

## Figures and Tables

**Table 1:** SPIRIT (Standard Protocol Items: Recommendations for Interventional Trials) schedule of enrolment, interventions, and assessment.

Timepoint	Study Period										
	Enrolment	Allocation	Post-Allocation								Close-Out
	2/2026–3/2026	2/2026–3/2026	Week 1	Week 2	Week 3	Week 4	Week 5	Week 6	Week 7	Week 8	5/2026
Enrolment											
Screening	√										
Eligibility	√										
Informed consent	√										
Allocation		√									
Intervention morning group			√	√	√	√	√	√	√	√	
Intervention evening group			√	√	√	√	√	√	√	√	
Control group			√	√	√	√	√	√	√	√	
Assessments											√
Health-related physical fitness		√								√	

**Table 2:** Detail protocol.

Time	Movements
0:00–0:40	High Knees
0:40–1:00	Rest
1:00–1:40	Jumping lunges
1:40–2:00	Rest
2:00–2:40	Jumping Jacks
2:40–3:00	Rest
3:00–3:40	Bodyweight Squats
3:40–4:00	Rest
4:00–4:40	Burpees
4:40–5:00	Rest
5:00–5:40	Butt kicks
5:40–6:00	Rest
6:00–6:40	Push ups
6:40–7:00	Rest
7:00–7:40	Mountain Climbers
7:40–8:00	Rest

**Table 3:** Interview guide.

No.	Interview Question
1.	Please describe your experience participating in the HIIT course.
2.	What was the most memorable movement for you throughout the entire module? Why?
3.	During the training, what specific physical changes did you notice? (e.g., fatigue level, heart rate, amount of sweating, muscle sensation)
4.	What impact do you think the HIIT has had on your physical fitness, endurance, or overall physical condition?
5.	How did you typically feel emotionally after participating in HIIT? (e.g., excited, stressed, focused, bored).
6.	During or after the training, did you experience any changes in your sense of accomplishment, motivation, self-confidence, or willpower? Please describe specifically
7.	Were there moments when you felt anxious, apprehensive, or wanted to give up? How did you cope with them?
8.	Do you think the current structure, duration, intensity, and movement arrangement of the HIIT module are reasonable? Why?
9.	What do you think are the advantages and potential disadvantages of this training method, respectively?
10.	Regarding safety, what areas do you think require special attention or need more guidance?
11.	Do you have any suggestions?

## Data Availability

The data presented in this study are available on request from the corresponding author. The data are not publicly available due to ethical restrictions.

## Abbreviations

The following abbreviations are used in this manuscript:

HIIT	High-intensity interval training
SPIRIT	Standard Protocol Items: Recommendations for Interventional Trials
MEQ	Morningness–Eveningness Questionnaire
IPAQ	International Physical Activity Questionnaire
BMI	Body mass index
1RM	One-repetition maximum
TCTR	Thai Clinical Trials Registry
